# Clinicopathologic features and treatment outcomes of dermatofibrosarcoma protuberans: a 25-year retrospective study^؆^

**DOI:** 10.1016/j.abd.2025.501213

**Published:** 2025-10-27

**Authors:** Lanyu Sun, Mafalda Pinho, Cláudia Brazão, Joana Frade, Diogo de Sousa, Pedro de Vasconcelos, Luís Soares-de-Almeida, Paulo Filipe

**Affiliations:** a Dermatology Service, Unidade Local de Saúde Santa Maria, Lisboa, Portugal; b Anatomical Pathology Service, Unidade Local de Saúde Santa Maria, Lisboa, Portugal; c Faculdade de Medicina da Universidade de Lisboa, Lisboa, Portugal; d Instituto de Medicina Molecular João Lobo Antunes, Lisboa, Portugal

**Keywords:** Dermatofibrosarcoma protuberans, Radiotherapy, Adjuvant, Skin neoplasms, Treatment outcome

## Abstract

**Background:**

Dermatofibrosarcoma protuberans (DFSP) is a rare cutaneous sarcoma characterized by slow growth, a high local recurrence rate, and low metastatic potential. *Objective:* To characterize the clinicopathological features and the treatment outcomes of patients diagnosed with DFSP.

**Methos:**

Retrospective study of patients with a histopathological diagnosis of DFSP between 1997 and 2022.

**Results:**

Data from 42 patients with DFSP were included. The majority were female (69%) with a mean age of 49.1 years. The trunk (52%) and extremities (40%) were the most common tumor sites. Classic histologic pattern was observed in 90% of cases, while rare variants, including fibrosarcomatous, pigmented, and myxoid subtypes, were also identified. Wide local excision was the primary treatment (95%), achieving clear margins in 71% of patients. Local recurrence occurred in 4.8% and adjuvant radiotherapy was employed in 19% of cases. Two patients developed pulmonary metastases with disease progression despite treatment with imatinib.

**Study limitations:**

Retrospective study based on medical and pathological records.

**Conclusions:**

Cutaneous DFSP in this series demonstrated clinicopathologic features consistent with those reported in the literature, including a predilection for the trunk and middle-aged females. Histologically, most cases exhibited the classic storiform pattern with strong CD34 positivity. Wide local excision was the primary treatment modality, with a relatively low recurrence rate compared to the literature, possibly influenced by the use of adjuvant radiotherapy in select patients. Although uncommon, metastasis occurred in cases with recurrent disease and fibrosarcomatous transformation. Ongoing research into systemic therapies and molecularly targeted treatments is needed to improve outcomes in advanced DFSP.

## Introduction

Dermatofibrosarcoma protuberans (DFSP) is a rare tumor, occurring at a rate of 0.8 to 4.5 cases per million persons annually and, despite being the most common cutaneous sarcoma, it constitutes less than 0.1% of all malignancies and about 1.0% of all soft tissue sarcomas worldwide.^1^, ^2^ It mainly affects young and middle-aged adults, with a peak of incidence between the second and fifth decades of age.^3^ Because DFSP is a slow-growing tumor, the diagnosis is often delayed for months to years.^2^ Despite its slow growth and infrequent distant metastasis, it carries a high risk of local recurrence,^4^, ^5^ emphasizing the importance of early diagnosis and appropriate treatment. This study aims to characterize the clinical and histopathological features of patients diagnosed with DFSP at a tertiary hospital over 25-years.

## Methods

This was a single-institution, retrospective, observational study of patients diagnosed with dermatofibrosarcoma protuberans (DFSP) between January 1997 and December 2022. Eligible cases were identified through a search of the institutional electronic medical record system and pathology database. Patients were included if they had a biopsy-confirmed diagnosis of DFSP and received treatment at the academic tertiary care hospital during the study period. Cases lacking complete medical or pathological data were excluded. Clinical information was extracted from medical charts and included sex, age at diagnosis, tumor location, clinical presentation, history of previous malignancies, treatment modalities, surgical margin status, use of adjuvant therapy, and clinical outcomes, including recurrence and metastasis. Follow-up time was calculated from the date of diagnosis to the last recorded clinical visit or patient death. Histopathological data were reviewed from archived Hematoxylin and Eosin (H&E) ‒ stained slides. Immunohistochemical staining for CD34 was reviewed when available. Descriptive statistics were used to summarize demographic, clinical, and pathological characteristics.

## Results

**Fig. 1:**
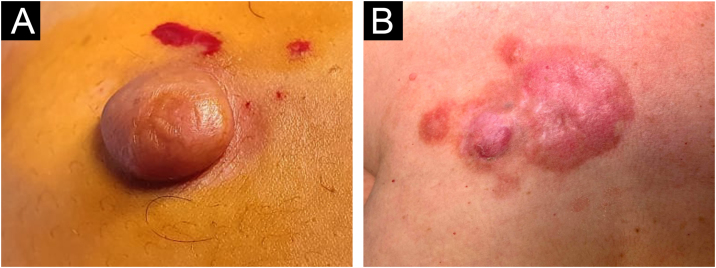
Clinical presentation of dermatofibrosarcoma protuberans. (A) Solitary, firm, dome-shaped nodule with a smooth surface. (B) Irregular, violaceous plaque with multiple nodular components.

**Fig. 2:**
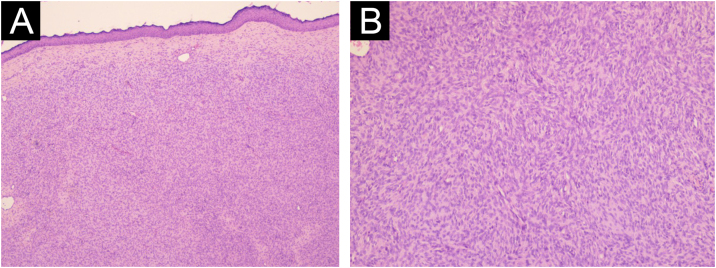
Histologic features of classic-pattern dermatofibrosarcoma protuberans: uniform spindle cells arranged in a storiform pattern infiltrate the dermis and extend into the subcutaneous tissue (A – Hematoxylin & eosin, ×40; B ‒ Hematoxylin & eosin, ×100).

**Fig. 3:**
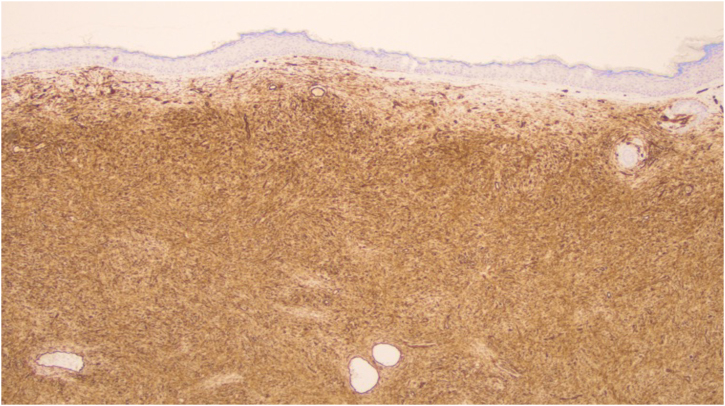
Immunohistochemical staining for CD34 showing strong and diffuse positivity in tumor cells (×40).

**Fig. 4:**
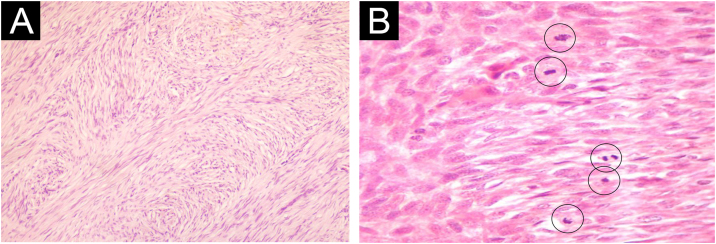
Dermatofibrosarcoma protuberans with fibrosarcomatous transformation. (A) Spindle-shaped cells arranged in intersecting bundles with a herringbone pattern (Hematoxylin & eosin, ×100). (B) Numerous mitoses are observed (highlighted with circles; Hematoxylin & eosin, ×400).

**Fig. 5:**
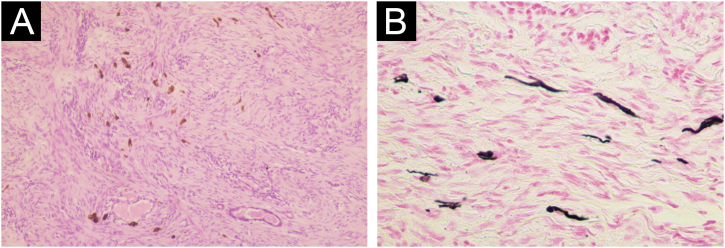
Pigmented dermatofibrosarcoma protuberans (Bednar tumour). (A) Numerous pigmented dendritic cells interspersed among spindle cells, demonstrating heavy melanin deposition (Hematoxylin & eosin, ×100). (B) The Fontana-Masson stain highlights the presence of melanin pigment within dendritic melanocytes interspersed among the spindle cell proliferation (Fontana-Masson, ×400).

**Fig. 6:**
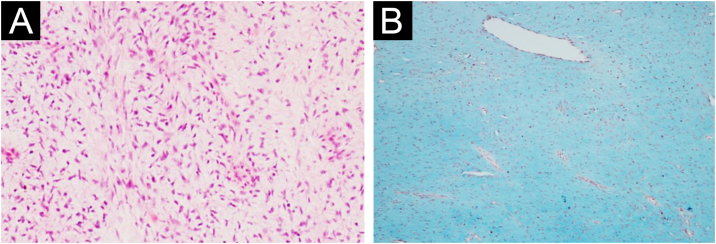
Dermatofibrosarcoma protuberans with myxoid change. (A) Myxoid spindle cell neoplastic proliferation in the dermis (Hematoxylin & eosin, ×100). (B) the prominent myxoid stroma, stains diffusely blue with Alcian blue (Alcian blue stain ×100).

A total of 42 patients with histologically confirmed dermatofibrosarcoma protuberans (DFSP) were included in this study (Table 1). The majority of patients were female (69%), and the mean age at diagnosis was 49.1-years (range: 22–78 years), with 55% of cases occurring in individuals under 50-years of age. Approximately 26% of patients had a prior history of other malignancies, most commonly breast cancer (10%) and melanoma (7%). The most frequent anatomical location of DFSP lesions was the trunk (52%), followed by the upper and lower extremities (40%). The head and neck region accounted for a minority of cases (7%). Clinically, DFSP manifested most often as an exophytic nodule (33%) (Fig. 1A), but also as plaques (19%) (Fig. 1B) and subcutaneous nodules (17%). Most patients (95%) presented with primary DFSP, while only two cases involved recurrent disease at initial evaluation. The classical histopathologic pattern was observed in 90% of cases, characterized by a storiform arrangement of uniform spindle cells infiltrating the dermis and subcutaneous tissue (Fig. 2). Immunohistochemical staining for CD34 showed strong and diffuse positivity in all cases (Fig. 3). Rare histologic variants were identified in 11% of cases, including fibrosarcomatous transformation (7%) (Fig. 4), pigmented (2%) (Fig. 5), and myxoid (2%) (Fig. 6) subtypes. Wide local excision (WLE) was the mainstay of treatment in 95% of cases, and clear resection margins (R0) were achieved in 71% of patients. Among the three cases of fibrosarcomatous DFSP, the American Joint Committee on Cancer (AJCC) / Union for International Cancer Control (UICC) stages were IA (T1N0M0G1), II (T1N0M0G2), and IV (T1N0M1G3). Adjuvant radiotherapy was administered in 19% of cases, predominantly in those with close or positive surgical margins. During a mean follow-up period of 10.6-years (range: 1–22 years), four patients experienced recurrence: two presented with disease recurrence at diagnosis, and two developed local recurrence following surgical treatment. All recurrent cases occurred in male patients, three of whom were aged 50-years or older, two had a history of positive resection margins, and the other two achieved complete resection with margins of 1 mm and 2 mm. One of these patients had fibrosarcomatous DFSP. Pulmonary metastases occurred in two patients ‒ one with a history of multiple local recurrences and another with fibrosarcomatous transformation. Both were treated with imatinib mesylate, and one also received sunitinib malate. Both patients died from disease progression.

**Table 1:** Patient demographics, clinical presentation, histopathological features, treatment, and outcomes.

Variable	No patients (%)
Age	
< 50 years	23 (55)
≥ 50 years	19 (45)
Mean (SD)	49.1 (±17.5)
Female sex	29 (69)
History of cancer	11 (26)
Location	
Trunk	22 (52)
Upper extremity	9 (21)
Lower extremity	8 (19)
Head and neck	3 (7)
Clinical feature	
Exophytic nodule	14 (33)
Plaque	8 (19)
Subcutaneous nodule	7 (17)
Not described	13 (31)
Clinical presentation	
Primary disease	40 (95)
Recurrent disease	2 (5)
Tumor size (cm)	
> 5	16 (38)
≤ 5	7 (17)
Not described	16 (38)
Lesion duration prior to diagnosis (years)	
> 5	8 (19)
≤ 5	7 (17)
Not described	27 (64)
Histopathological subtype	
Classic	37 (90)
Fibrosarcomatous	3 (7)
Pigmented (Bednar tumour)	1 (2)
Myxoid	1 (2)
COL1A1-PDGFB fusion protein	
Detected by FISH	2
Not performed	40
Treatment	
Wide local excision	40 (95)
Mohs micrographic surgery	2 (5)
Resection margins	
Free (R0)	31 (74)
Positive	11 (26)
Number of surgeries to achieve negative margins	
1	29 (69)
2	10 (24)
3	3 (7)
Adjuvant radiotherapy	8 (19)
Adjuvant systemic therapy	2 (5)
Distant metastasis	2 (5)
Death due to DFSP	2 (5)

## Discussion

Dermatofibrosarcoma protuberans (DFSP) is an uncommon, low-grade sarcoma of fibroblast origin.^6^ The predominance of DFSP in females (69%) observed in this study aligns with some reports suggesting a slight female preponderance,^7^ although other studies report a more balanced gender distribution.^5^ The mean age of diagnosis at 49.1 years reflects the typical presentation in middle adulthood, although DFSP can occur at any age, including in pediatric populations.^7^

Patients with DFSP, especially females, had a 25% increased risk of developing a subsequent primary malignancy, with most of the increased risk owing to a greater than 21-fold increased risk of other non-epithelial skin cancer and a nearly 5-fold increased risk of soft tissue cancer.^8^ Approximately 26% of the studied patients had a history of other malignancies.

Clinically, DFSP presents as a slow-growing, firm, multilobular nodule or plaque that ranges in color from flesh-coloured to red and has irregular margins.^9^ Most DFSPs are localized on the trunk (40%–50%), followed by proximal extremities (30%–40%), and the head and neck (10%–15%).^2^, ^7^ The clinical features of the studied patients were consistent with previous results, except for the location in the head and neck which was less frequent than in other series (7%).

Histologically, DFSP tumor cells present classically as uniform spindle cells arranged in a characteristic well-defined storiform pattern;^6^ they have minimal cytologic atypia, uniform nuclei, and absent-to-low mitotic activity.^10^ Strong CD34 positivity remains a hallmark diagnostic feature and serves to differentiate DFSP from benign entities such as dermatofibroma.^2^ Rare histological variants were also observed in the present series. Fibrosarcomatous DFSP (FS-DFSP) is defined by the focal presence of areas with fascicles of spindle cells intersecting at acute angles in a typical “herringbone” pattern. In these areas, the mitotic activity is increased, and the cells show mild to moderate cellular and nuclear pleomorphism, and necrosis can be found.^5^ This subtype of DFSP is associated with a more aggressive clinical course, higher recurrence rates, and increased metastatic potential.^6^, ^11^ Of the two cases with pulmonary metastasis, one exhibited the fibrosarcomatous variant.

More than 90% of DFSP cases are associated with a t(17;22)(q22;q13) translocation, resulting in the COL1A1-PDGFB fusion protein.^7^ Molecular testing, such as Reverse Transcription Polymerase Chain Reaction (RT-PCR) or Fluorescence In Situ Hybridization (FISH), is available to detect this translocation.^2^ In the present study, FISH was performed on only two patients, as it was not available at the studied institution.

Complete resection is the primary treatment for DFSP. The modern options include wide local excision (WLE), mohs micrographic surgery (MMS), and surgery followed by three-dimensional complete circumferential and peripheral deep margin assessment (or CCPDMA).^10^ Achieving clear margins is critical, as positive margins are a well-documented risk factor for local recurrence.^9^ Tumor cells tend to invade the subcutaneous tissue with irregular tentacle-like projections through septa and fat lobules. Therefore, local recurrences after excision with apparently wide margins are common.^5^ Some studies propose that MMS may result in lower local recurrence rates than WLE for DFSP. Notably, DFSP treated with WLE exhibits a recurrence rate that ranges from 10% to 60%,^6^ contrasting with the 1% recurrence rate observed with MMS treatment.^12^, ^13^ However, MMS and its variants are a time-consuming technique, and not widely diffused as a standard surgery procedure. For such reasons, several authors have proposed WLE (with at least 3 cm including the underlying fascia) as a valid therapeutic option to MMS.^9^, ^14^ Most patients (95%) at the studied institution underwent WLE for treatment, as MMS is not available at the facility. The local recurrence rate after definitive therapy in the clinics was 4.8%, which is inferior to the literature data with WLE but higher than MMS. In the present series, this lower recurrence rate may be attributed to the approach of achieving wide surgical margins whenever feasible and the use of adjuvant radiotherapy for cases with narrow or positive margins.

Because DFSP is relatively sensitive to radiation, radiation therapy may be an important treatment option for inoperable tumors or when postoperative margins are positive, or for tumors with multiple recurrences.^9^, ^10^ Radiotherapy has been suggested to reduce the risk of local recurrence after inadequate surgical resection in patients with narrow or positive surgical margins.^15^ Adjuvant radiotherapy was employed in 19% of the present cases, primarily for patients with positive resection margins. The authors hypothesize that this may have contributed to the lower recurrence rate after surgery observed in this series compared to the literature, regardless of WLE.

The clinicopathologic features reported in the literature as being associated with disease recurrence include a high-grade component (fibrosarcomatous change, increased cellularity, and high mitotic rate), close to a positive surgical margin, patient age > 50 years, a large tumor size (≥ 5 cm), male sex, and a location on the head, neck, or extremity rather than on the trunk.^4^, ^15^, ^16^ Since the authors recorded only four patients with recurrent disease, the capacity to characterize the impact of clinicopathologic features on recurrence after surgery is limited.

The variable time to recurrence (1–14 years) observed in this study underscores the importance of long-term follow-up for DFSP patients. European guidelines recommend follow-up examinations every 6-months for the first 5-years and at yearly intervals thereafter for up to 10-years. Surveillance strategies should include regular clinical examinations and MRI monitoring for patients with deeply invasive disease or a high risk of recurrence.^6^, ^17^

The overall risk for the development of metastatic disease is 5%, including 1% with regional lymph node metastasis and 4% with distant metastasis. The lung is the most common site of metastasis.^2^ The two cases of pulmonary metastases highlight the clinical challenges of managing advanced DFSP. These cases suggest that recurrent disease and fibrosarcomatous transformation may increase the risk of metastasis. Tyrosine kinase inhibitors have shown promising response rates in patients with advanced or metastatic disease.^2^ Both patients were treated with imatinib mesylate, a Tyrosine Kinase Inhibitor (TKI) targeting the PDGFB fusion protein.^10^ While imatinib has revolutionized the management of advanced and metastatic DFSP, resistance can develop, necessitating the exploration of alternative agents such as sunitinib or second-generation TKIs.^18^ Despite systemic therapy, both patients died due to disease progression, underscoring the need for continued research into novel therapeutic strategies, including immunotherapy and targeted molecular approaches.

This study has limitations, including its retrospective design and incomplete clinical data in some cases, especially those diagnosed further in the past. Additionally, the lack of MMS at the studied institution limited direct comparison with WLE outcomes.

## Conclusion

Cutaneous DFSP in this series demonstrated clinicopathologic features consistent with those reported in the literature, including a predilection for the trunk and middle-aged females. Histologically, most cases exhibited the classic storiform pattern with strong CD34 positivity. Wide local excision was the primary treatment modality, with a relatively low recurrence rate compared to the literature, possibly influenced by the use of adjuvant radiotherapy in selected patients. Despite its generally favorable prognosis, the potential for late local recurrence and metastatic disease supports the need for vigilant, long-term follow-up. The adverse clinical course associated with advanced DFSP underscores the limited efficacy of current systemic therapies and highlights the critical role of early diagnosis, complete surgical excision, and adjuvant radiotherapy. Ongoing research into novel systemic and molecularly targeted treatments is essential to improve outcomes in late-stage DFSP.

## ORCID ID

Mafalda Pinho: 0000-0003-2644-9343

Cláudia Brazão: 0000-0002-4485-7124

Joana Frade: 0000-0002-5471-7889

Pedro de Vasconcelos: 0000-0002-7480-1228

Luís Soares-de-Almeida: 0000-0003-4026-6105

Paulo Filipe: 0000-0002-7337-6493

## Financial support

None declared.

## Authors’ contributions

Lanyu Sun: Study concept and design; data collection, analysis, and interpretation; manuscript writing; critical review of important intellectual content and the literature; active participation in research supervision; and approval of the final version of the manuscript.

Mafalda Pinho: Data collection, analysis, and interpretation; manuscript writing; critical review of important intellectual content and the literature; and approval of the final version of the manuscript.

Cláudia Brazão: Data collection, analysis, and interpretation; critical review of important intellectual content and the literature; and approval of the final version of the manuscript.

Joana Frade: Data collection, analysis, and interpretation; critical review of important intellectual content and the literature; and approval of the final version of the manuscript.

Diogo de Sousa: Study concept and design; data collection, analysis, and interpretation; and approval of the final version of the manuscript.

Pedro de Vasconcelos: Data collection, analysis, and interpretation; critical review of important intellectual content; and approval of the final version of the manuscript.

Luís Soares-de-Almeida: Study concept and design; critical review of important intellectual content; active participation in research supervision; and approval of the final version of the manuscript.

Paulo Filipe: Study concept and design; critical review of important intellectual content; active participation in research supervision; and approval of the final version of the manuscript.

## Research data availability

The entire dataset supporting the results of this study was published in this article.

## Conflicts of interest

None declared.
